# The Prevention of Eating Disorders in Australian Adolescents: A Modeled Cost‐Effectiveness Study

**DOI:** 10.1002/eat.70048

**Published:** 2026-01-30

**Authors:** Long Khanh‐Dao Le, Eng Joo Tan, Phillipa Hay, Jaithri Ananthapavan, Yong Yi Lee, Cathrine Mihalopoulos

**Affiliations:** ^1^ Health Economics Group, School of Public Health and Preventive Medicine Monash University Melbourne Victoria Australia; ^2^ Translational Health Research Institute (THRI), School of Medicine Western Sydney University Penrith New South Wales Australia; ^3^ Camden and Campbelltown Hospital SWSLHD Campbelltown New South Wales Australia; ^4^ Deakin Health Economics, Institute for Health Transformation, School of Health and Social Development Deakin University Burwood Victoria Australia; ^5^ Global Centre for Preventive Health and Nutrition, Institute for Health Transformation, School of Health and Social Development Deakin University Burwood Victoria Australia; ^6^ School of Public Health The University of Queensland Herston Queensland Australia; ^7^ Queensland Centre for Mental Health Research Wacol Queensland Australia

**Keywords:** adolescents, body project, eating disorder, prevention

## Abstract

**Objective:**

Prevention programs for eating disorders (EDs) have the potential to reduce the onset of these diseases and improve the mental health and well‐being of the general population. However, there is mixed evidence on whether routine implementation of such programs at the population level is cost‐effective. This study intends to investigate the cost‐effectiveness of an evidence‐based preventive intervention for EDs, the Body Project, at the population level.

**Method:**

The Body Project is a targeted school‐based intervention that aims to prevent EDs among adolescents. A Markov model was developed to evaluate the incremental cost‐effectiveness of the hypothetical implementation of the Body Project among female‐identifying secondary students in Australia versus a ‘no intervention’ comparator. A cost‐utility analysis was conducted from a “healthcare and limited education” sector perspective with costs and health impacts modeled over the lifetime of the target population. An incremental cost‐effectiveness ratio (ICER), expressed as cost per health‐adjusted life year (HALY) gained, was calculated. Sensitivity analyses were done to test model assumptions.

**Results:**

If implemented across 1416 Australian secondary schools, the modeled Body Project consisting of four group sessions for eligible girls aged 15–18 years with high body image concerns was estimated to yield about 92 HALYs (95% CI: 58–131) and save $2.56 million in future healthcare costs at an implementation cost of $1.88 million (95% CI: $1.62–$2.15 million).

**Discussion:**

The Body Project intervention is likely to represent good value for money. Successful implementation of this program will require further research into its feasibility and acceptability among schools and the wider community.

## Key Points

- *Cost‐effective and potentially cost‐saving*: The Body Project, a school‐based intervention targeting adolescent girls with body image concerns, was found to be cost‐saving with healthcare offsets and cost‐effective even without them, with an incremental cost‐effectiveness ratio (ICER) of $20,504 per health‐adjusted life‐years (HALY) gained, which is well below common willingness‐to‐pay thresholds in Australia.
- *Positive health and economic outcomes*: Modeled over the lifetime of the target population, the program prevented an estimated 221 new cases of eating disorders and gained 92 HALYs on average, while saving $2.56 million in healthcare costs.
- *Implementation challenges*: Despite strong evidence of effectiveness and safety, uncertainties remain regarding program acceptability, feasibility, long‐term sustainability, and equity.
- *Robust findings*: Sensitivity analyses confirmed the robustness of results across multiple scenarios, including variations in intervention duration, population reach, and effectiveness across different subgroups.

## 1 Introduction

Eating disorders (EDs) are serious mental health conditions that affect millions of people worldwide (Erskine et al. 2016). EDs are characterized by persistent disturbances in eating behavior and body image, resulting in significant impairment in physical and psychological functioning (American Psychiatric Association 2013). EDs have the highest mortality rate among all psychiatric disorders (Arcelus et al. 2011), as well as high rates of morbidity, comorbidity (usually with depression and anxiety), and reduced quality of life (Tan et al. 2023). Moreover, EDs impose a substantial economic burden on individuals, families, and society, due to direct healthcare costs and productivity losses (Ágh et al. 2016).

The onset of EDs typically occurs during adolescence and young adulthood, a critical period of development and transition (Javaras et al. 2015). Adolescence is also a time when body dissatisfaction, dieting, and disordered eating behaviors are prevalent among young people (Larson et al. 2021). Therefore, adolescence represents a window of opportunity for the prevention of EDs and the promotion of positive body image and healthy eating behaviors. Effective and scalable preventive interventions can reduce the incidence and prevalence of EDs and improve the mental health and well‐being of the population (Wilksch and Wade 2017).

Preventive interventions for EDs aim to reduce the risk factors and enhance the protective factors associated with the development and maintenance of ED symptoms (Le et al. 2023). A range of preventive interventions across the age spectrum and different population groups have been developed and evaluated, such as psychoeducation, media literacy, cognitive‐behavioral therapy (CBT), and peer‐led approaches (Watson et al. 2016). A majority of these interventions were shown to be effective in mitigating ED risk factors, although there was variability in the reported effect sizes across different intervention techniques and settings (Le et al. 2023). A recent systematic review and meta‐analysis of ED prevention interventions found that the most promising approach was dissonance‐based interventions, which involve challenging the internalization of sociocultural ideals of thinness and beauty (Le, Barendregt, Hay, and Mihalopoulos 2017).

The most widely implemented and researched dissonance‐based intervention is the Body Project (Dakanalis et al. 2019), a group‐based program that targets female adolescents and young women (Stice 2002). Several randomized controlled trials and meta‐analyses have demonstrated the efficacy and effectiveness of the Body Project in reducing ED risk factors, symptoms, and onset across diverse settings and populations (Stice et al. 2013, 2021; Becker et al. 2016). Despite the strong evidence for the effectiveness of the Body Project, there is limited evidence on the economic credentials of this program, particularly on the cost‐effectiveness of the program's implementation at a population level. For example, a previous modeling study suggested that the Body Project may not be cost‐effective when delivered to the eligible population in Australia (Le, Barendregt, Hay, Sawyer, et al. 2017). In contrast, a study that modeled a clinical‐trial population of 149 young women estimated that a virtual version of the Body Project was cost‐effective in Sweden (de Martínez Alva et al. 2023).

Moreover, none of these studies modeled the cost‐effectiveness of the Body Project over a longer term period (> 10 years) and very few investigated its implementation and scalability across the general population. Cost‐effectiveness modeling uses mathematical simulations to estimate the long‐term health outcomes and costs of delivering an intervention compared with not delivering it. By combining data on how programs will be implemented in a specific country context and associated costs, changes in disease risk, and quality‐of‐life gains, these models show whether an intervention represents good value for money in a specific setting. It provides an assessment of whether the cost of implementing an intervention or program is worth the benefits it produces. Because costs, healthcare prices, and population risks differ between countries, the same program may appear more or less cost‐effective depending on context.

In short, cost‐effectiveness modeling can help decision‐makers weigh the likely benefits, costs, and trade‐offs of alternative prevention options, supporting efficient and equitable use of limited public resources (World Health Organization 2011).

The aim of this study was to model the long‐term cost‐effectiveness of the hypothetical implementation of the Body Project program across all secondary schools in Australia. A comprehensive and validated model that incorporates the effects of ED symptoms and diseases on health outcomes and costs will be used to model the impact of this intervention on Australian adolescents. The current study was conducted within the context of a larger research project that assessed the cost‐effectiveness of high BMI and ED prevention interventions in Australia (ACE‐HiBED) (Le et al. 2020).

## 2 Methods

The present analysis focuses on adolescent girls because the Body Project was originally developed and evaluated for female participants, with strong evidence of efficacy in this group (Dakanalis et al. 2019; Stice 2002; Stice et al. 2013, 2021; Becker et al. 2016). Epidemiological data consistently show that EDs are more prevalent among female‐identifying populations, with adolescent girls experiencing two to three times higher rates of body dissatisfaction and disordered eating behaviors than adolescent boys (Smink et al. 2012; Mitchison and Hay 2014). While gender‐diverse young people are also at markedly elevated risk for EDs (Nagata et al. 2020; Watson et al. 2017), there remains insufficient intervention and modeling data to accurately estimate program reach or effectiveness for these populations.

The ACE‐HiBED model was used to estimate the cost‐effectiveness of implementing the Body Project intervention among Australian adolescents. In brief, this model uses mathematical simulations to follow a large, representative population over time, comparing what would happen with and without the intervention in terms of health outcomes and costs. This model was developed by repurposing an existing model, the ACE‐Obesity Policy model (Ananthapavan et al. 2020), to include ED symptoms as a risk factor and several EDs as diseases of interest. Model validation was supported through several factors. First, the ACE‐Obesity Policy model has been used extensively to evaluate the economic credentials of a range of obesity prevention programs and policies (Lal et al. 2017; Ananthapavan et al. 2019). Second, the inclusion of the ED model component was done in consultation with subject experts in ED and obesity, who were part of the Project Steering Committee for the ACE‐HiBED project. This model has been used to evaluate the Health Weight, a preventive intervention for both EDs and obesity in those with high body image concerns (Le et al. 2024).

A cost‐utility analysis framework was adopted. This type of analysis compares the additional costs of an intervention with the additional health gains it produces, expressed as a combined measure of length and quality of life known as health‐adjusted life years (HALYs) (Moreno‐Ternero et al. 2023). The disability weights of ED were sourced from an extended Global Burden of Disease (GBD) study estimating the global prevalence and burden of binge‐ED and other specified feeding or EDs (Santomauro et al. 2021). For the purpose of this study, three major types of EDs were considered—anorexia nervosa (AN), bulimia nervosa (BN), and binge‐eating disorder (BED). A “health and limited education” sector perspective was used, meaning that both healthcare and education system costs, as well as potential savings to the healthcare sector, were included. Potential benefits to the education sector from this intervention were not included in the analysis. All costs and cost offsets were converted to 2019 Australian dollars using health price deflators (Australian Institute of Health and Welfare 2022). A discount rate of 3% per annum, used in similar studies in the Australian setting (Lal et al. 2017; Ananthapavan et al. 2019), was applied to all costs and benefits. The main results were reported as incremental cost‐effectiveness ratios (ICERs). The ICER represents how much extra it costs to gain one additional HALY from the intervention compared with doing nothing. The intervention was assessed as representing good value for money if the ICER was below the willingness‐to‐pay threshold of $50,000 per HALY gained, which has been used in previous Australian economic evaluation studies (Carter et al. 2008). As an alternative, a range of willingness‐to‐pay thresholds as outlined in the Productivity Commission Inquiry into Mental Health was also considered: < $33,000 (very cost‐effective), < $64,000 (cost‐effective), and < $96,000 (marginally cost‐effective) (Australian Government. Productivity Commission 2020). This study adheres to the Consolidated Health Economic Evaluation Reporting Standards (CHEERS) guidelines, as detailed in Data S1 (Husereau et al. 2013).

### 2.1 Intervention Description

The Body Project intervention is a selective prevention program that is based on cognitive dissonance principles and targets young women with body image concerns. Through a series of facilitated group discussions, participants who have internalised the thin‐ideal voluntarily engaged in verbal, written, and behavioral exercises in which they critiqued this ideal (e.g., write essays and conduct role‐plays that are counter‐attitudinal). These activities should lead to psychological discomfort in participants and motivate them to reduce thin‐ideal internalisation, which will then decrease body dissatisfaction, dieting, negative affect, and ED symptoms. The intervention is delivered by trained facilitators (usually clinical graduate students) using a group discussion format (6–10 participants) through four sessions with each session lasting 60 min (Stice et al. 2009; Shaw and Stice 2016). For the current evaluation, we modelled the intervention as being delivered in schools with at least one existing school counsellor.

### 2.2 Eligible Population for the Intervention

Data from previous Body Project effectiveness trials as outlined in Shaw and Stice (2016) were used to model the number of persons from the population who would be eligible to undertake the intervention in this hypothetical implementation of the Body Project in Australia. The following assumptions were made in our modeled evaluation of the hypothetical implementation of the Body Project. The modeling included all female students aged 15–18 who are enrolled in secondary schools recruited through emails, flyers, and leaflets. These recruitment materials invite females with body image concerns to participate in the Body Project intervention. The modeling assumed that the proportion of participants who responded to these recruitment materials was estimated to be approximately 10%, based on a previous Body Project clinical trial, would participate in an Australian program with a similar intervention pathway (Shaw and Stice 2016). After telephone screening, students who verbally affirmed having high body image concerns would have been offered the intervention. According to the 2013 Mission Australia Youth Survey of young women (Perrens et al. 2013), the proportion of Australian female students who self‐identify as having high body image concern is about 68.9%. Analysis of similar randomized controlled trials (RCT) indicates that 83% (range: 64%–95%) of students agreed to participate in the intervention after the initial screening; in this study, a more conservative estimate of 45% was applied to account for real‐world implementation challenges of program delivery in every high school in Australia (Le, Barendregt, Hay, Sawyer, et al. 2017). In 2019, approximately 570,000 females aged 15–18 years would have been eligible for the Body Project intervention.

### 2.3 Effectiveness of Intervention

A meta‐analysis of eight Body Project studies estimated that the intervention was effective in reducing ED symptoms compared to waitlist control at post‐test (Hedges' *g* = −0.30, 95% confidence interval [CI]: −0.47 to −0.13) with substantial heterogeneity (*I* ^2^ = 51%, *Q* = 16.49, *p* = 0.04) (Le, Barendregt, Hay, and Mihalopoulos 2017). At 1‐year follow up, the intervention effect on ED symptoms persisted although it was not statistically significant (Hedges' *g* = −0.06, 95% CI −0.19 to 0.06, *I* ^2^ = 0%, *Q* = 4.76, *p* = 0.45). Based on these effects (including the CIs to account for uncertainty), a fitted asymptotic function was used to estimate the decay rate of intervention effect on ED symptoms from 1‐year to 10‐year post intervention. Zero intervention effect is assumed in subsequent years. Using a methodology outlined in Lee et al. (2018), the Hedges' g effect sizes on ED symptoms were converted to relative risk (RR) effect sizes (for the incidence of ED disease). The RR effect size is commonly used in decision‐analytic models of health care interventions to calculate the change in disease‐related incidence and/or remission following an intervention. Applying this method, we estimated that the Body Project intervention can reduce 15% of the incidence of AN, BN and BED post‐intervention (RR = 0.85, 95% CI: 0.79–0.91).

### 2.4 Costs

The costs associated with the intervention included the expenses for project administration, recruitment and screening, school counselor training, and the time spent by school counselors to deliver the intervention. The unit costs for a national‐level project coordinator and school counselors were obtained from the Australian Bureau of Statistics (ABS) Employee Earnings report and a 30% on‐costs were added (to account for leave entitlements, superannuation, other benefits and overheads) (Australian Bureau of Statistics 2023). The cost of training school counselors was based on the Body Project facilitator training program offered by Eating Disorders Victoria (Eating Disorders Victoria 2023). The time and travel expenses of students were not taken into consideration as the intervention takes place in a school setting and would thus incur zero marginal cost to students. The intervention was assumed to be delivered in a “steady state” operation, that is, trained staff and necessary infrastructure were available to scale up the intervention, which was assumed to be working in accordance with their efficacy potential (World Health Organization 2011; Australian Institute of Health and Welfare 2022). Annual costs for the management of AN, BN, and BED were estimated using data from a recent population‐based study investigating the economic costs of ED in Australia (Tannous et al. 2022). EDs costs included hospital costs, out‐of‐hospital costs (including GP services, imaging, pathology, psychologists, and medical specialists), and medication costs. Table 1 lists further information, including the key assumptions and cost items included in the delivery of the Body Project intervention.

**TABLE 1:** Assumption and cost items for the Body Project intervention.

Parameters	Assumption/unit cost	Sourced
Recruitment process	A school counselor spent 10 min to introduce and explain flyers to 25 students (equivalent to one secondary‐school class size)	Expert opinion
Screening process	A school counselor spent 10 min (5 min preparation + 5 min phone call) to do a screening per student	Expert opinion
Training	Each school receives formal training (for one school counselor) from Body Project experts (10 h training plus 15 min of technical support)	Expert opinion
School counselors (average salary of school counselors)	$1532 per week; $40.90 per hour (assuming 37.5 h of work per week) + 30% on‐costs	Australian Bureau of Statistics Employee Earnings and Hours Report, 2021
One project coordinator (average salary of policy and planning manager)	$3300 per week; $87.99 per hour (assuming 37.5 h of work per week) + 30% on‐costs	Australian Bureau of Statistics Employee Earnings and Hours Report, 2021
Training cost		
Online training	$416 per training	Eating Disorders Victoria (EDV)'s Body Project Facilitator Training Program
Technical assistance (4 h)	$125 per hour	
On‐costs	30%	Expert opinion

### 2.5 Modeling Health Outcomes

**FIGURE 1:**
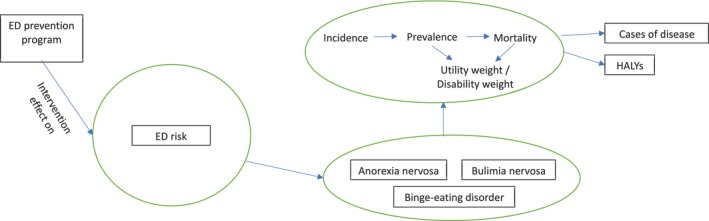
Schematic representation of the ACE‐HiBED model.

The ACE‐HiBED model is a simulation tool built in Microsoft Excel 2010 that estimates how much better or worse population health and costs would be if an ED prevention program were implemented compared with doing nothing. While the change in risk of developing an ED was modeled over a 10‐year period, the model continued to follow those simulated individuals over their entire lifetime (up to 10 years) to capture long‐term health and cost consequences. The model determines the number of health‐adjusted life years (HALYs) gained by estimating how many healthy years of life are gained if fewer people developed or lived with ED because of the intervention. It does this by comparing the incidence, prevalence and mortality rates of ED between two simulated populations (one receiving the intervention and one not receiving it). A diagram of the model is displayed in Figure 1. In the model, reducing ED symptoms lowers the number of new cases (incidence), which over time decreases the total number of people living with an ED (prevalence), as well as related deaths and disability. These improvements translate into more healthy life years and lower healthcare costs. For the purpose of this study, only the ED‐specific component of the ACE‐HiBED model was used to assess the cost‐effectiveness of the Body Project, as this intervention targets body image and disordered‐eating symptoms rather than BMI.

Each ED was modeled using four health states, namely “healthy,” “diseased,” “dead due to the disease,” and “dead due to other causes.” The age‐ and sex‐specific probabilities of transitioning between health states were calculated using the DisMod II software (Barendregt et al. 2003). Epidemiology‐related model inputs such as total population prevalent years lived with disability (Vos et al. 2012), incidence, prevalence, and mortality of the diseases were taken from the GBD 2019 study (Vos et al. 2020). The impact of the diseases on health was measured using the years lived with disability multiplied by the disease‐specific disability weights, sourced from the GBD studies (Le et al. 2024; Salomon et al. 2012).

The AN, BN, and BED incidence rates from GBD 2019 were adjusted in the model to account for age and risk factors related to body dissatisfaction. Research has shown that higher levels of body dissatisfaction increase the likelihood of developing an ED at the ages of 15 and 16, with odds ratios of 1.51 and 1.67, respectively (Rohde et al. 2015). These odds were transformed into RRs of 1.44 and 1.57 (Barendregt 2012). An assumption was made that the RR of 1.57 would also apply to those aged between 17 and 100 years old and was used to adjust the incidence of ED for this age group. HALYs were calculated by aggregating the population level changes to overall mortality and morbidity for each ED (using the disability weights).

### 2.6 Uncertainty and Sensitivity Analysis

Uncertainty analysis using Monte Carlo simulation was performed to assess the robustness of results to parameter uncertainty. The simulation incorporated probability distributions associated with the input parameters of the model, RRs, disability weights, and costs. The model was re‐run and the values drawn from the defined distribution parameters (5000 bootstrap replications) to obtain estimates of incremental costs and HALYs, using the Ersatz bootstrap add‐in software for Microsoft Excel. Details of the parameters varied in the uncertainty analysis are presented in Table 2. The results of the uncertainty simulations were presented on a cost‐effectiveness plane, which plots cost differences against benefit differences compared to the “no intervention” comparator. Interventions located above the horizontal axis are more costly while those to the right of the vertical axis are more effective (relative to the comparator).

**TABLE 2:** Input parameters and uncertainty ranges used in the modeling.

Parameters	Values and uncertainty range	Uncertainty distributions	Source
Efficacy (RR of developing ED)		Lognormal	From a meta‐analysis of four RCTs (Le et al. 2022)
Post test	0.85 (95% CI: 0.79–0.92)		
1‐year follow up	0.94 (95% CI: 0.86–1.03)		
Epidemiological parameters for AN, BN and BED diseases (per person‐year): Incidence Prevalence Mortality	By age group (see Source)	Lognormal	Santomauro et al. (2021)
All‐cause mortality rate (per 100,000)	By age group (see Source)	N/A	Santomauro et al. (2021)
Body dissatisfaction adjusted for incidence (RR)		Lognormal	Rohde et al. (2015)
Age 15	1.44 (1.00–2.05)		
Age 16 and above	1.58 (1.17–2.13)		
Disability weight		Beta	Santomauro et al. (2021)
Anorexia nervosa	0.22 (95% CI: 0.15–0.31)		
Bulimia nervosa	0.22 (95% CI: 0.15–0.31)		
Binge‐eating disorder	0.05 (95% CI: 0.02–0.08)		
Private costs (including unit costs of school counselors, screening materials and training costs)	±20%	Pert	Own assumption
Number of sessions	4	Discrete	From four RCTs
Make‐up session duration (hour)	0.2 (range: 0.15–0.25)	Pert	From four RCTs
2019 Australian female population	15 years: 140,400 16 years: 140,297 17 years: 140,910 18 years: 147,688 Total: 569,295	N/A	Australian Bureau of Statistics (2020)
Proportion of population who responded to recruitment materials (%)	10 (range: 9–12)	Pert	Stice et al. (2009)
Proportion of population who self‐identified as having high body image concerns (%)	68.9 (range: 20–80)	Pert	Perrens et al. (2013)
Proportion of eligible population who accepted the intervention (%)	83 (range: 64–95)	Pert	From 12 RCTs
Average treatment cost (per incidence/prevalent case): Anorexia nervosa Bulimia nervosa Binge‐eating disorder	By age group (see Source)	Lognormal	Tannous et al. (2022) Australian Institute of Health and Welfare (2022)

Abbreviations: CI, confidence interval; ED, eating disorder; RCTs, randomized controlled trials; RR, relative risk; WMD, weighted mean difference.

In addition to uncertainty analysis, a series of one‐way sensitivity analyses were performed to examine the impact of different plausible assumptions and parameters on the cost‐effectiveness results. The following scenarios were modeled:

Scenario 1: Intervention effect decay rate of 50% per annum. No intervention effect after 10 years.

Scenario 2: The intervention did not prevent AN (informed by the lack of consensus in the literature on whether existing preventive interventions were effective for AN prevention) (Becker et al. 2016).

Scenario 3: The target population included males aged 15–18 years old. The same intervention effect was applied.

Scenario 4: The target population was expanded to include younger girls and young female adults (12–25 years old) with the assumption that the intervention had the same intervention effect in this broader population. With this scenario, we also assumed that instead of a school counselor, a university counselor or nurse could conduct the screening.

Scenario 3 explored a hypothetical extension of the Body Project to include male adolescents. Due to the lack of robust evidence on intervention effectiveness in males, the same effect size observed in females was assumed as a simplifying and transparent assumption. This scenario was intended as an exploratory sensitivity analysis to examine potential implications for scalability and inclusivity, rather than to imply equivalent effectiveness across genders.

### 2.7 Implementation Considerations

Although this study primarily examined the cost‐effectiveness of the hypothetical implementation of the Body Project program, there are additional factors that can impact the routine adoption of the program. These factors, known as “implementation considerations” (Carter et al. 2008), are not reflected in the technical cost‐effectiveness results, but impact decision‐making. The study analyzed several implementation considerations, such as strength of evidence supporting the effectiveness of the program, acceptability of the program to stakeholders including parents, students and schools, feasibility of implementation, equity, sustainability (i.e., how the intervention can be delivered in the long term rather than environmental sustainability), safety, and other potential secondary effects. These implementation considerations were identified through extensive consultation with the ACE‐HiBED Project Steering Committee, which consisted of clinical experts and stakeholders including representatives from National Eating Disorders peak bodies and the National Mental Health Commission. A “positive” rating for an implementation consideration favors intervention implementation; “uncertain” indicates it neither supports nor weakens the case for intervention implementation; and “negative” indicates it does not support intervention implementation.

## 3 Results

**FIGURE 2:**
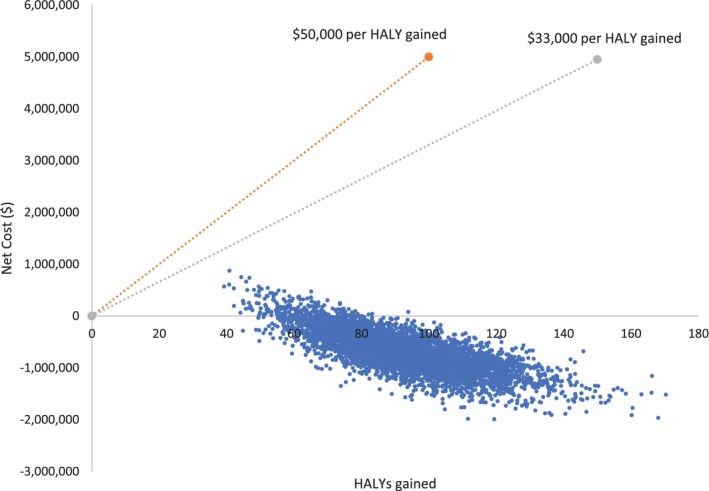
Cost‐effectiveness plane of base case analysis with willingness‐to‐pay threshold of $50,000 and $33,000 per HALY.

Table 3 presents the economic evaluation results of the hypothetical implementation of the Body Project intervention. If implemented across 1416 secondary schools in Australia (and delivered to approximately 17,000 eligible female students who would have agreed to participate in the intervention), the cost of intervention implementation was $1.88 million (95% CI: $1.62–$2.15 million), which translated to approximately $117 per student (i.e., per participant in the eligible population). The intervention led to a reduction of 28, 87, and 106 incidence cases of AN, BN, and BED, respectively. The mean incremental HALYs gained was 92 (95% CI: 58–131), while healthcare cost savings were estimated to be $2.56 million (95% CI: $1.73–$3.41 million), and the net costs were −$0.68 million (95% CI: −$1.45 to $0.08 million). The cost‐effectiveness plane shows that a majority of the runs of the model (96%) fell in the south‐east quadrant, which means that the intervention was generally more effective and less costly than no intervention (Figure 2). Without cost‐offsets, the mean ICER for this intervention was $20,504 per HALY gained (95% CI: $16,378–$27,920), and none of the iterations fell above the willingness‐to‐pay threshold of $50,000 per HALY. In relation to a secondary willingness‐to‐pay threshold of $33,000 per HALY gained, 98% of model runs resulted in an ICER (without cost‐offsets) being cost‐effective.

**TABLE 3:** Results of cost‐effectiveness analysis for the Body Project intervention.

	Mean	95% CI	Probability to be cost‐effective at $50,000 per HALY gained	Probability to be cost‐effective at $33,000 per HALY gained
HALYs gained	92.45	57.92–131.10	—	—
Cost (exclude cost‐offsets)	$1,880,378	$1,617,171–$2,147,105	—	—
Cost (include cost‐offsets)	−$679,706	−$1,450,592 to $83,717	—	—
ICER per HALY gained (exclude cost‐offsets)	$20,504	$16,378–$27,920	100.0%	97.6%
ICER per HALY gained (include cost‐offsets)	Dominant	Dominant to $1364	100.0%	100.0%

*Note*: Dominant indicates that the intervention is more effective and less costly than the comparator group.

Abbreviations: HALY, health‐adjusted life years; ICER, incremental cost‐effectiveness ratio.

### 3.1 Sensitivity Analysis

The evaluation was the least sensitive to an expanded female target population (Scenario 4) while for the remaining scenarios the Body Project intervention yielded a positive ICER (more costly but more effective) if cost‐offsets were included. For example, in scenario 1, the ICER was $39,764 (95% CI: $28,768–$66,644) per HALY gained if there was a 50% annual decay of intervention effect and no cost‐offsets included. The intervention was also 78% likely to be cost‐effective at willingness‐to‐pay threshold of $50,000 per HALY gained. In Scenario 2, the assumption that the intervention did not prevent AN led to slightly higher ICER ($29,450 per HALY gained, 95% CI: $22,775–$41,179) compared to the baseline scenario but it was still lower than the willingness‐to‐pay threshold. Similarly, including males aged 15–18 years old as part of the target population in Scenario 3 increased the ICER slightly to $30,119 (95% CI: $24,179–$40,470) when cost offsets were excluded. This increase was primarily due to lower healthcare cost savings associated with ED prevention for males in comparison to females. In the final scenario, assuming no cost‐offsets, the ICER was $16,123 (95% CI: $12,549–$21,469) per HALY gained and the intervention was cost‐effective at all examined willingness‐to‐pay thresholds when the target population was expanded to include females aged 12–25 years old. Further details of sensitivity analysis were presented in Table 4.

**TABLE 4:** Results of one‐way sensitivity analyses.

	Mean	95% CI	Probability to be cost‐effective at $50,000 per HALY gained	Probability to be cost‐effective at $33,000 per HALY gained
Scenario 1: 50% decay rate				
HALYs gained	47.95	24.19–74.81	—	—
Cost (exclude cost‐offsets)	$1,881,432	$1,612,427–$2,152,130	—	—
Cost (include cost‐offsets)	$564,322	Dominant to $1,198,852	—	—
ICER (exclude cost‐offsets)—per HALY gained	$39,764	$28,768–$66,644	78.2%	25.0%
ICER (include cost‐offsets)—per HALY gained	$14,884	Dominant to $48,184	97.8%	92.4%
Scenario 2: AN not prevented				
HALYs gained	64.82	39.30–94.43	—	—
Cost (exclude cost‐offsets)	$1,884,582	$1,618,347–$2,150,702	—	—
Cost (include cost‐offsets)	$79,422	Dominant to $611,135	—	—
ICER (exclude cost‐offsets)—per HALY gained	$29,450	$22,775–$41,179	98.1%	67.9%
ICER (include cost‐offsets)—per HALY gained	$1961	Dominant to $13,988	100.0%	99.9%
Scenario 3: Male population included				
HALYs gained	116.43	73.85–164.68	—	—
Cost (exclude cost‐offsets)	$3,483,538	$2,988,829–$3,981,816	—	—
Cost (include cost‐offsets)	$404,094	Dominant to $1,329,273	—	—
ICER (exclude cost‐offsets) – per HALY gained	$30,119	$24,179–$40,470	98.2%	65.9%
ICER (include cost‐offsets) – per HALY gained	$4283	Dominant to $16,998	100.0%	99.8%
Scenario 4: Female target population expanded				
HALYs gained	391.46	248.71–567.86	—	—
Cost (exclude cost‐offsets)	$6,227,024	$5,339,554–$7,126,153	—	—
Cost (include cost‐offsets)	($4,359,362)	Dominant	—	—
ICER (exclude cost‐offsets)—per HALY gained	$16,123	$12,549 to $21,469	100.0%	99.6%
ICER (include cost‐offsets)—per HALY gained	Dominant	Dominant	100.0%	100.0%

*Note*: Dominant indicates that the intervention is more effective and less costly than the comparator group.

Abbreviations: AN, anorexia nervosa; HALY, health‐adjusted life years; ICER, incremental cost‐effectiveness ratio.

### 3.2 Implementation Considerations

Table 5 presents the results of the implementation considerations. Positive ratings were given for strength of evidence, safety, and potential secondary effects. For example, as reported by several meta‐analyses, there is evidence to support the Body Project in terms of prevention of EDs. Similarly, the evaluation did not consider long‐term educational and/or productivity outcomes and likely underestimated the full benefits arising from preventive interventions. Uncertain ratings were given for acceptability and feasibility. For example, the acceptability of the intervention is uncertain because it is dependent on the type of stakeholder and may elicit mixed responses from schools (negative), students (uncertain), and government bodies (positive), while the feasibility of implementing the intervention requires significant commitment from school counselors and potentially teachers. A negative rating was given for sustainability given the large degree of uncertainty around the long‐term financial support for the Body Project program. Similarly, a negative rating was assigned to equity because while females generally have a higher risk of disease of ED compared to males, targeted interventions such as the Body Project, both genders should have access to effective interventions that prevent ED.

**TABLE 5:** Implementation considerations arising from the Body Project intervention.

Implementation consideration	Rationale for overall rating	Overall rating
Strength of evidence	There is strong evidence to support Body Project in terms of prevention of eating disorders.	Positive
Acceptability	There is an uncertainty around acceptability because it is likely dependent on the type of stakeholder. Coeducational schools may hesitate to implement this intervention since it was primarily developed for female participants and participation rate among high school students can be low due to lack of interest and competing with other mental health prevention program. Although some form of support is likely from the government, there may be confusion regarding jurisdiction and responsibilities between state and federal governments.	Uncertain
Feasibility	The Body Project intervention is short and delivered in a group format. Further discussions are needed to evaluate whether counselors are willing to attend training workshops which are available both online and in person. In addition, questions around enrolment and attrition need to be addressed as well as support administration from the schools' perspective. Training feasibility across high schools in Australia needs to be considered. Another consideration is the presentation of school counselors in every secondary school who will conduct screening phase of the intervention.	Uncertain
Sustainability	A large degree of uncertainty surrounds the implementation and operation of the program in the long term. In particular, it is not very clear which entity will be providing financial resources to support the program and whether existing infrastructure are sufficient to deliver the intervention at scale.	Negative
Equity	The primary target population of the Body Project intervention was adolescent and young adult females only. As such, boys in schools are potentially excluded or may not receive suitable intervention content.	Negative
Safety	There were no issues or unintended consequences reported by the various randomized controlled trials that implemented the healthy weight intervention.	Positive
Potential secondary effects	The economic evaluation did not include an examination of other potential effects, such as academic performance changes and career prospects in adulthood. Evidence suggests that people with ED have a higher risk of developing mental health problems. Therefore, the prevention of ED is likely to increase productivity (and reduce healthcare utilization) and therefore this approach undertaken is conservative and likely to undervalue the benefits arising from the intervention.	Positive

## 4 Discussion

In this study, a population‐based Markov model (ACE‐HiBED) was developed to estimate the cost‐effectiveness of the hypothetical implementation of the Body Project intervention for the prevention of ED. This study demonstrated that, using several commonly accepted willingness‐to‐pay thresholds, Body Project is likely to be cost‐saving (i.e., more effective and less costly) than no intervention and was cost‐effective even when healthcare cost‐savings were excluded. The sensitivity analysis demonstrated that variations in some of the modeling parameters did not change the results significantly.

The findings in this study are consistent with those reported in another modeled economic evaluation of a virtual‐based Body Project (vBP) program to prevent ED in young women in Sweden (de Martínez Alva, et al. 2023). The authors found that the vBP program yielded lower cost and higher health benefits (expressed as quality‐adjusted life‐years, QALYs) compared to a do‐nothing alternative and, hence, resulted in a dominant ICER. Similar findings were also observed in several trial‐based economic evaluations of the Body Project. For example, in a study of 408 young women recruited from eight U.S. universities, the authors concluded that the Body Project was likely to be of economic value with a cost of US$838 for each individual with reduced ED symptoms (Akers et al. 2017). In another trial‐based study with a slightly larger sample size of 680 young women in the U.S., the authors reported a cost of US$740 for each additional case of ED prevented by the Body Project program (Akers et al. 2021). Our study also updates a previous study using a population‐based Markov model with Australian female adolescents, in which the Body Project was not considered cost‐effective with an ICER of $103,980 per disability‐adjusted life‐year (DALY) averted (Le, Barendregt, Hay, Sawyer, et al. 2017). First, our model was based on underlying epidemiology data that is ED‐specific (e.g., AN, BN) whereas the previous model input in Le, Barendregt, Hay, and Mihalopoulos (2017) was based on an amalgamation of AN and BN epidemiological data. Therefore, it is likely that the amalgamated data have underestimated the incidence and/or prevalence of AN and BN in the baseline population. Second, the model in Le, Barendregt, Hay, and Mihalopoulos (2017) did not include BED in their classification of ED due to data unavailability from burden of disease study at this time, which potentially underestimated the healthcare cost savings obtained from the prevention of this type of ED.

The results of this study make a significant contribution to the growing research on the cost‐effectiveness of prevention programs for ED. An analysis of implementation considerations also revealed several positive aspects of the Body Project intervention such as strength of evidence, safety, and potential secondary effects. Nevertheless, successful implementation of this intervention in a real‐world setting and at scale would require further evaluation of its acceptability, feasibility, sustainability, and equity impacts. For example, it is necessary to have further discussions with various stakeholders such as school staff, parents, community organizations, and health professionals about integrating the Body Project program within the school curriculum and identifying any potential unintended consequences arising from its implementation.

The strength of the current study is the use of plausible assumptions based on the latest available literature to develop the economic model. However, there are several limitations. First, our evaluation only considered costs that were within the health and education sectors. While education‐sector implementation costs were included, the analysis did not capture potential downstream cost offsets within schools, such as reduced need for student support services or improved educational outcomes. This represents a limited education‐sector perspective and may underestimate the broader economic benefits of preventive programs delivered in educational settings. It is also known that EDs are associated with higher costs outside the health sector. For example, in a study of a subsample of ED patients (university students) from a treatment‐based trial, it was found that up to 40% of the costs associated with EDs are attributable to indirect costs (or productivity costs) (Javaras et al. 2015). Therefore, it is likely that our evaluation underestimated the potential cost‐savings from the prevention of ED, which can emerge later in the life span. Second, our evaluation was conducted to assess the cost‐effectiveness of ED prevention from an Australian perspective that may not be generalizable to other countries. For example, the proportion of female adolescents who have high body image concerns and those who receive the intervention may be different across countries. Another limitation relates to gender inclusivity. The model was based on data from trials conducted with cisgender female participants, reflecting the original target group of the Body Project. As such, findings may not generalize to boys or gender‐diverse adolescents, highlighting the need for future research on adapting and evaluating prevention programs that are inclusive of all genders within school settings. In addition, the delivery of the Body Project program may vary due to differences in education systems between countries.

## 5 Conclusion

This study suggests that the Body Project program delivered to Australian female adolescents with body image concerns to prevent ED is likely to be more effective and less costly relative to the “no intervention” control. From a healthcare system perspective, the intervention was found to be cost‐saving over the longer term and represents good value for money. Further research on Body Project's feasibility and acceptability among schools and the wider community is necessary to ensure its successful implementation at the population level.

## Author Contributions

**Long Khanh‐Dao Le:** conceptualization (equal), data curation (equal), formal analysis (equal), funding acquisition (lead), investigation (equal), methodology (equal), project administration (supporting), writing – original draft preparation (equal), writing – review and editing (equal). **Eng Joo Tan:** conceptualization (equal), data curation (equal), formal analysis (equal), investigation (equal), methodology (equal), project administration (lead), visualization (lead), writing – original draft preparation (equal), writing – review and editing (equal). **Phillipa Hay:** conceptualization (supporting), formal analysis (supporting), funding acquisition (equal), investigation (supporting), methodology (supporting), supervision (equal), validation (equal), writing – review and editing (equal). **Jaithri Ananthapavan:** conceptualization (supporting); formal analysis (supporting); funding acquisition (equal); investigation (supporting); methodology (supporting); supervision (equal); validation (equal); writing – review and editing (equal). **Yong Yi Lee:** conceptualization (supporting), formal analysis (supporting), funding acquisition (equal), investigation (supporting), methodology (supporting), supervision (equal), validation (equal), writing – review and editing (equal). **Cathrine Mihalopoulos:** conceptualization (supporting), formal analysis (supporting), funding acquisition (equal), investigation (supporting), methodology (supporting), supervision (equal), validation (equal), writing – review and editing (equal).

## Funding

This work was supported by the National Health and Medical Research Council, APP1183225.

## Disclosure

Use of AI was not involved in any component of preparation of this manuscript. Persons with lived experience were not involved in the study design or execution, or in the preparation of this manuscript.

## Ethics Statement

The authors have nothing to report.

## Conflicts of Interest

The authors declare no conflicts of interest.

## Supporting information


**Data S1:** eat70048‐sup‐0001‐Supinfo.docx.

## Data Availability

The data that support the findings of this study are available from the corresponding author upon reasonable request.
